# Supplementary material for: From sequence to enzyme mechanism using multi-label machine learning
Source: BMC Bioinformatics. 2014 May 19;15:150. doi: 10.1186/1471-2105-15-150 (PMC4229970; doi:10.1186/1471-2105-15-150)
Supplement: Additional file 2 — Java code of ml2db. Additional file ml2db_code.tar.gz contains the Java source code to run the multi-label machine learning experiments and save the results to database. The code’s Javadoc is included. [file 1471-2105-15-150-S2.zip › additional file 2/ml2db/ml2db/doc/mulan/classifier/neural/model/ActivationLinear.html]

ActivationLinear


JavaScript is disabled on your browser.


- Overview
- Package
- Class
- Use
- Tree
- Deprecated
- Index
- Help

- Prev Class
- Next Class

- Frames
- No Frames

- All Classes

- Summary:
- Nested |
- Field |
- Constr |
- Method

- Detail:
- Field |
- Constr |
- Method


mulan.classifier.neural.model

## Class ActivationLinear

- java.lang.Object
- - mulan.classifier.neural.model.ActivationFunction
  - - mulan.classifier.neural.model.ActivationLinear

- All Implemented Interfaces:
  :   java.io.Serializable

  ---

    

  ```
  public class ActivationLinear
  extends ActivationFunction
  ```

  Implements the linear activation function. The input is simply passed to the output.
  This activation function is commonly used for input units of networks, which serves
  as a place holders for input pattern and forwards them for processing.

  Version:
  :   2012.02.27

  Author:
  :   Jozef Vilcek

  See Also:
  :   Serialized Form

- - ### Constructor Summary

    Constructors

    Constructor and Description

    `ActivationLinear()`
  - ### Method Summary

    Methods

    | Modifier and Type | Method and Description |
    | --- | --- |
    | `double` | `activate(double input)` Computes an output value of the function for given input. |
    | `double` | `derivative(double input)` Computes an output value of function derivation for given input. |
    | `double` | `getMax()` Gets the maximum value the function can output. |
    | `double` | `getMin()` Gets the minimum value the function can output. |

    - ### Methods inherited from class java.lang.Object

      `equals, getClass, hashCode, notify, notifyAll, toString, wait, wait, wait`

- - ### Constructor Detail


    - #### ActivationLinear

      ```
      public ActivationLinear()
      ```
  - ### Method Detail


    - #### activate

      ```
      public double activate(double input)
      ```

      **Description copied from class: `ActivationFunction`**

      Computes an output value of the function for given input.

      **Specified by:**
      :   `activate` in class `ActivationFunction`

      Parameters:
      :   `input` - the input value to the function

      Returns:
      :   the output value


    - #### derivative

      ```
      public double derivative(double input)
      ```

      **Description copied from class: `ActivationFunction`**

      Computes an output value of function derivation for given input.

      **Specified by:**
      :   `derivative` in class `ActivationFunction`

      Parameters:
      :   `input` - the input value to the function

      Returns:
      :   the output value


    - #### getMax

      ```
      public double getMax()
      ```

      **Description copied from class: `ActivationFunction`**

      Gets the maximum value the function can output.

      **Specified by:**
      :   `getMax` in class `ActivationFunction`

      Returns:
      :   maximum value of the function


    - #### getMin

      ```
      public double getMin()
      ```

      **Description copied from class: `ActivationFunction`**

      Gets the minimum value the function can output.

      **Specified by:**
      :   `getMin` in class `ActivationFunction`

      Returns:
      :   minimum value of the function


- Overview
- Package
- Class
- Use
- Tree
- Deprecated
- Index
- Help

- Prev Class
- Next Class

- Frames
- No Frames

- All Classes

- Summary:
- Nested |
- Field |
- Constr |
- Method

- Detail:
- Field |
- Constr |
- Method
